# 3D printing-based full-scale human brain for diverse applications

**DOI:** 10.1002/brx2.5

**Published:** 2023-04-05

**Authors:** Weijian Hua, Cheng Zhang, Lily Raymond, Kellen Mitchell, Lai Wen, Ying Yang, Danyang Zhao, Shu Liu, Yifei Jin

**Affiliations:** 1 Department of Mechanical Engineering, University of Nevada Reno, Reno, Nevada, USA; 2 School of Mechanical Engineering, Dalian University of Technology, Dalian, Liaoning, China; 3 Department of Pharmacology, Center for Molecular and Cellular Signaling in the Cardiovascular System, School of Medicine, University of Nevada, Reno, Nevada, USA; 4 Department of Chemistry, University of Nevada Reno, Reno, Nevada, USA; 5 Department of Gerontology, The First Affiliated Hospital of China Medical University, Shenyang, Liaoning, China

**Keywords:** 3D printing, brain patch, full-scale human brain models, stimuli-responsive yield-stress bath, surgery training, wound healing

## Abstract

Surgery is the most frequent treatment for patients with brain tumors. The construction of full-scale human brain models, which is still challenging to realize via current manufacturing techniques, can effectively train surgeons before brain tumor surgeries. This paper aims to develop a set of three-dimensional (3D) printing approaches to fabricate customized full-scale human brain models for surgery training as well as specialized brain patches for wound healing after surgery. First, a brain patch designed to fit a wound’s shape and size can be easily printed in and collected from a stimuli-responsive yield-stress support bath. Then, an inverse 3D printing strategy, called “peeling-boiled-eggs,” is proposed to fabricate full-scale human brain models. In this strategy, the contour layer of a brain model is printed using a sacrificial ink to envelop the target brain core within a photocurable yield-stress support bath. After crosslinking the contour layer, the as-printed model can be harvested from the bath to photo crosslink the brain core, which can be eventually released by liquefying the contour layer. Both the brain patch and full-scale human brain model are successfully printed to mimic the scenario of wound healing after removing a brain tumor, validating the effectiveness of the proposed 3D printing approaches.

## 1 | INTRODUCTION

Primary malignant brain tumors, such as glioblastoma multiforme,^[1,2]^ are the tenth leading cause of death for human beings. In 2020, approximately 85,000 people were diagnosed with brain tumors, and more than 700,000 people had to live with a primary malignant brain tumor in the United States.^[3–5]^ Although treatment for brain tumors is affected by various factors, including type, location and size of a tumor, patient’s age and health, and so on, surgery is still the most frequent option for patients with brain tumors.^[6,7]^ Brain tumor surgery is a neurosurgical procedure that involves the removal of abnormal tissues from a brain with specific surgery instruments, suturing of the incision, and postoperative recovery.^[8,9]^ Preoperative preparation is crucial before the tumor resection due to the complexity of the brain’s architecture. Patient-targeted surgery training can effectively enhance the success rate of the upcoming surgery, which firstly requires reliable manufacturing techniques to construct a full-scale human brain model based on the patient’s computed tomography (CT) scanning data. In the postoperative recovery stage, materials or drugs are necessary to restore the incision, especially skin tissue damage around the scalp or skull, and to clear any residual tumor cells. Bioactive nanomaterials have been extensively employed in wound healing,^[10–12]^ circulating tumor cell capture,^[13]^ and tumor immunotherapy.^[14]^ Postoperative patches can be constructed from nanomaterials or loaded with nanomaterials to effectively enhance wound healing and tumor cell clearance.

Three-dimensional (3D) printing is capable of fabricating customized products with complex geometries.^[15,16]^ Of the diverse 3D printing techniques, material extrusion has been widely used because of its unique merits, such as easy implementation, a wide range of printable materials, and relatively high printing speed, to name a few.^[17–19]^ Particularly, yield-stress support bath-assisted 3D printing, an emerging material extrusion strategy, has received increasing attention in recent years.^[20–23]^ In this strategy, a liquid 3D structure is printed in a yield-stress bath, which provides ubiquitous mechanical supports during printing. After printing, corresponding crosslinking mechanisms are applied to cause the solidification of the printed structure, making it releasable from the bath. Although yield-stress support bath-assisted 3D printing presents broader fabrication capability, engineered full-scale human brains have not been printed using this technique. For example, Hinton et al.^[21]^ developed a method called freeform reversible embedding of suspended hydrogels (FRESH), in which gelatin microparticles were used to prepare a yield-stress support bath. Using this method, they created a 3D brain structure from alginate which was scaled down to 3 mm in length. Jin et al.^[22]^ used the nanoclay suspension as the yield-stress support bath to print a compressed human brain model, which had a scaled-down ratio of 1:10, from gelatin. There are two main engineering issues in full-scale brain model printing. First, current support bath materials^[23–27]^ possess poor flowability at working status due to their high yield stresses (ranging from tens to hundreds of Pascals^[20,22,28,29]^), making it extremely challenging to add support bath materials during printing and eventually constraining the overall size of achievable structures^[22,29]^ as well as ink selection.^[20,28]^ Second, the human brain is composed of dense tissue. Directly printing a full-scale human brain within a support bath is not only time-consuming (an estimated printing period of ~30 h in total for 100% infill) but also difficult to control the volume of necessary support bath material because the model being printed keeps occupying the space in the support bath. A recently developed stimuli-responsive yield-stress fluid-assisted 3D printing approach^[30]^ is promising to overcome the first issue by on-demand adding bath materials during printing, but it is still invalid for the second issue. Furthermore, it is still unclear whether the proposed approach can practically realize the creation of full-scale human tissues and organs. To improve printing efficiency, Zhao et al.^[31]^ reported an inverse 3D printing approach, in which a silicone oil ink was used to draw the contour of a target structure in a photo crosslinkable support medium. After crosslinking, the support medium was mechanically stripped to release the target structure along the silicone oil contour. Nevertheless, due to the difficulty of adding support medium during printing, only a scaled-down brain model (30% of the full-scale human brain) was printed. Moreover, this mechanical stripping process may damage the target structure, especially when the structure has complex geometries. Finally, the stripped crosslinked support medium is not recyclable, leading to the generation of material waste in each fabrication cycle. Therefore, it calls for the development of new support bath-assisted 3D printing strategies.

## 2 | METHODS

### 2.1 | Materials preparation

#### 2.1.1 | Material preparation for patch printing

The nanoclay-Pluronic F127 yield-stress support bath was prepared according to the protocol outlined in the previously published paper.^[30]^ First, a nanoclay suspension was prepared by mixing the required amount of Laponite^®^ RD powder (BYK Additives Inc.) with deionized (DI) water at the concentration of 2.0% (w/v) at room temperature (RT). The mixture was continuously stirred using an overhead stirrer (LACHOI) at 800 rpm for at least 40 min to fully hydrate the nanoclay powder. Next, an appropriate amount of Pluronic F127 powder (P2443, Sigma-Aldrich) was added to the nanoclay suspension at the concentration of 30.0% (w/v). Then, the nanocomposite was stirred at 4°C for a minimum of 60 min until the mixture became clear and homogeneous. To eliminate bubbles, the nanocomposite was degassed using a centrifuge (Cole-Parmer^®^ VS3400, Cole Parmer Instrument Company) at 2500 rpm for 2 min. The prepared material was stored in a sealed container at 4°C to prevent evaporation and aged for 1 day before use.

For the photocurable ink preparation, polyethylene glycol diacrylate (PEGDA) stock (Mn 700, Sigma-Aldrich) was diluted with DI water at a concentration of 6.0% (v/v). Then, 0.5% (w/v) of photo-initiator (2-hydroxy-4’-(2-hydroxyethoxy)-2-methylpropiophenone, Sigma-Aldrich) was added to the diluted PEGDA solution and thoroughly mixed until fully dissolved at RT. After that, an appropriate amount of sodium alginate powder (NaC_6_H_7_O_6_, Sigma-Aldrich) was dispersed into the solution at the concentration of 6.0% (w/v) and continually stirred using an overhead stirrer (Thermo Fisher Scientific) for 40 min at 700 rpm. The solution was degassed using the centrifuge at 2500 rpm for 2 min and loaded into a syringe as the photocurable ink. For rheological measurements, the 6% (v/v) PEGDA solution without alginate was prepared per the same protocol.

#### 2.1.2 | Material preparation for brain printing

For the preparation of a photocurable yield-stress support bath, a stock solution of PEGDA (Mn 700, Sigma-Aldrich) was diluted with DI water to the required concentration. Then, 0.5% (w/v) of the photoinitiator was added and mixed thoroughly. After that, the same protocol used for the nanoclay-Pluronic F127 nanocomposite was applied to prepare the photocurable yield-stress fluids at the nanoclay concentration of 2.0% (w/v) and Pluronic F127 concentration of 30.0% (w/v). Finally, the prepared nanocomposites were degassed, stored in a sealed container at 4°C, and aged for 1 day before use. In particular, the nanocomposites with different PEGDA concentrations (5.0%, 10.0%, and 20.0% (v/v)) were prepared for the rheological measurements, mechanical tests, and crosslinking depth characterization.

To prepare the sacrificial ink, an appropriate amount of gelatin powder (Emprove^®^ Essential, Sigma-Aldrich) was added into DI water at the concentration of 5.0% (w/v) and mixed with an overhead stirrer (Thermo Fisher Scientific) at 600 rpm for at least 30 min at 40°C. Then, sodium alginate powder was dispersed into the gelatin solution at the concentration of 6.0% (w/v) and stirred at 40°C and 800 rpm for 40 min. The prepared solution was degassed using the centrifuge at 3000 rpm for 2 min to remove any air bubbles and loaded into a syringe before use.

In addition, a 2.0% (w/v) calcium chloride (CaCl_2_) bath was prepared by dispersing the appropriate amount of CaCl_2_ powder (Sigma-Aldrich) into DI water and manually mixed until completely dissolved, which was used for ionically crosslinking the sacrificial ink. A sodium citrate solution was also prepared by dispersing the appropriate amount of sodium citrate powder (C_6_H_5_O_7_·2H_2_O·3Na, Sigma-Aldrich) into DI water at the concentration of 3.0% (w/v), which was applied for de-crosslinking the gelled contour layer.

### 2.2 | Material characterization

#### 2.2.1 | Rheological measurements

The rheological properties in this work were measured using a rheometer (MCR 92, Anton Paar) with a cone-plate geometry (at an angle of 1°, a diameter of 50 mm, and a cone-to-plate gap distance of 0.102 mm). The viscosities of the photocurable ink (6% (v/v) PEGDA and 6.0% (w/v) NaAlg) and 6.0% (v/v) PEGDA solution without alginate were measured via the steady shear rate sweeps at 25°C with the shear rate varying from 10^−2^ to 10^3^ s^−1^ for the photocurable ink and from 10^0^ to 10^3^ s^−1^ for the 6.0% (v/v) PEGDA solution, respectively. In addition, the steady shear rate sweeps were conducted at 4 and 25°C, respectively, to the photocurable yield-stress baths with different PEGDA concentrations (i.e., 5.0%, 10.0%, and 20.0% (v/v)) to determine their yield stress values. The shear rate was varied from 10^−2^ to 10^3^ s^−1^. The thermosensitivity of the sacrificial ink was characterized by the temperature sweeps, in which the shear rate was set at 1 s^−1^, while the temperature was increased from 5 to 50°C with an interval of 1°C. The viscosity change during the temperature increase was recorded.

#### 2.2.2 | Degradation of the photocurable ink

The specimens were made from the photocurable ink and cast in a cylinder model with dimensions *Φ*13 × 9 mm. They were then incubated in a phosphate-buffered saline (PBS) solution (pH 7.4 (1×), Gibco) at 37°C. The PBS solution was renewed every 2 days. The degradation process was monitored by calculating the mass loss ratio of the specimens over 14 days suing the formula as follows:

$$\text{Mass}\;\text{loss}\;\text{ratio}\;=\frac{W_{0}\;-\;W_{d}}{W_{0}}\times 100\%$$

where $W_{0}$ is the initial mass of the specimens and $W_{d}$ is the weighted mass on each measured day.

#### 2.2.3 | Mechanical property characterization

Uniaxial unconfined compression tests were performed on the cast cylindrical samples (*Φ*13 × 9 mm) at 25°C using a mechanical tester (eXpert 7600, ADMET). The strain rate was kept constant at 1.0 mm/min for all experiments. The stress-strain curve was determined based on the load and displacement data and the sample dimensions. Thus, the elastic modulus for each sample can be calculated from the curve. Particularly, the photocurable ink samples were first crosslinked using ultraviolet (UV) radiation provided by a UV curing system (OmniCure Series 2000, wavelength: 320–500 nm, Lumen Dynamics) for 10 min and then subjected to degradation in the PBS solution for 7 days before testing. The 6.0% (v/v) PEGDA samples were crosslinked using UV light for 10 min and tested thereafter. For brain printing, the photocurable yield-stress bath samples with varying concentrations of PEGDA (i.e., 5.0%, 10.0%, and 20.0% (v/v)) were crosslinked under UV radiation for 10 min and then tested.

#### 2.2.4 | UV transparency of the nanoclay-Pluronic F127 support bath

UV/VIS spectrophotometry was carried out using a UV-Vis spectrophotometer (Shimadzu^®^ UV-2550, Shimadzu Scientific Instruments) to determine the UV absorbance of the nanoclay-Pluronic F127 support bath for patch printing. The nanocomposite with 2.0% (w/v) nanoclay and 30.0% (w/v) Pluronic F127 was loaded in a rectangular quartz cuvette (10 mm path, Style C, Fisher Scientific), while DI water was used as a reference to measure the baseline. The absorbances of the nanocomposite were recorded via the wavelength sweeping from 300 to 500 nm.

#### 2.2.5 | Crosslinking depth of photocurable yield-stress support bath under UV radiation

The crosslinking depths of the photocurable yield-stress bath materials with different PEGDA concentrations were measured using the following procedure. First, the centrifuge tubes (15 mL, Membrane Solutions) were cut to have a height of ~51.0 mm, filled with the photocurable yield-stress bath materials at 4°C, and wrapped with aluminum foil. When the temperature had recovered to 25°C, the tubes were exposed to UV radiation provided by the UV curing system, which was positioned 5.0 cm above the tubes. Thus, the photo crosslinking process in the tubes was unidirectional. The exposure time was set to 5, 10, 20, 40, 60, 120, 240, 600, and 1200 s, respectively. The thickness at each exposure time was measured as the curing depth for each material in this work.

### 2.3 | Printing system and protocols

An extrusion 3D bioprinter (3D-Bioplotter Starter series, EnvisionTEC) was utilized to perform different printing experiments. To investigate the effects of path speed, the photocurable ink was extruded through a 25-gauge dispensing nozzle (inner diameter of 250 μm and length of 25.4 mm) (EFD Nordson) to generate filaments in the nanoclay-Pluronic F127 support bath. The dispensing pressure was set at 2 bar (2 × 10^5^ Pa), and the path speed was increased from 2 to 20.0 mm/s with an interval of 2.0 mm/s. The filaments at different path speeds were imaged, and their diameters were measured using ImageJ (https://imagej.nih.gov/ij/). To study the effects of step distance, straight cylindrical tubes (*Φ*10 mm) from the photocurable ink were printed using the same 25-gauge dispensing nozzle. The path speed was controlled at 12.0 mm/s to achieve filaments with a diameter of around 250 μm. The dispensing pressure was 2 bar. All tubes were printed for 40 layers but with different step distances of 150, 200, and 250 μm. A digital camera (120 fps USB Camera, ELP) was used to record the tube printing process. The height of each printed tube $\left(H_{m}\right)$ was measured using ImageJ, and the height ratio was calculated using the following equation:

$$\text{Height}\;\text{ratio}\;=\frac{H_{m}}{H_{D}}$$

where $H_{D}$ is the designed tube height, which is equal to step distance × layers.

### 2.4 | UV penetration and de-crosslinking of the sacrificial ink

To characterize the UV penetration through a solidified sacrificial ink layer, the photocurable yield-stress bath material at 4°C was filled into a 15 mL centrifuge tube to reach a depth of 50.0 mm. Then, the bath material switched to the solid state when the temperature was increased to 25°C. After that, the sacrificial ink was perfused into the tube with a depth of 1 mm on top of the bath material, which was further ionically crosslinked using CaCl_2_ and thermally gelled at 4°C for 30 min, resulting in a dual-crosslinked sacrificial ink layer. The tube was then wrapped in aluminum foil and placed 5 cm below the UV curing system for 20 min to crosslink the bath material. The thickness of the resulting crosslinked support bath material was used to quantify the depth of UV penetration. To study the de-crosslinking process, the sacrificial ink was cast into a homemade polydimethylsiloxane (PDMS) mold with a diameter of 32.0 mm and a depth of 1.0 mm. The mold was then submerged into a CaCl_2_ bath at 4°C for 30 min for ionic and thermal crosslinking. After that, the mold with the crosslinked sample was moved to a sodium citrate bath at 37°C. The time required for the sample to spread in the bath was recorded as the de-crosslinking time.

### 2.5 | 3D patch and brain model fabrication

The 3D bioprinter was used to fabricate both the 3D brain patch and the full-scale human brain model. For patch printing, a red food dye (McCORMICK & CO., INC.) was added to the photocurable ink at the concentration of 0.1% (v/v) and mixed thoroughly at 25°C to enhance visualization. The ink was then loaded into a 10 mL syringe (EFD Nordson), and the 25-gauge dispensing nozzle was applied for patch printing. The step distance, dispensing pressure, and path speed were set at 200 μm, 2 bar, and 12.0 mm/s, respectively. The photocurable ink was printed into a 3D patch structure in a transparent container (50 × 50 × 30 mm^3^) full of the nanoclay-Pluronic F127 nanocomposite. The printing process was conducted at 25°C. After printing, the patch structure was crosslinked by UV radiation for 10 min. Finally, the container was moved to a refrigerator at 4°C for 1 h to liquefy the support bath material. Thus, the solidified 3D brain patch can be harvested from the bath.

For the fabrication of the full-scale human brain model, a 20-gauge dispensing nozzle (EFD Nordson) with an inner diameter of 500 μm and a length of 12.7 mm was used to print the contour layer. The step distance, dispensing pressure, and path speed were set to 400 μm, 0.7 bar (0.7 × 10^5^ Pa), and 18.0 mm/s, respectively. The red food dye was added to the sacrificial ink at 0.1% (v/v) to visualize the printing process. The ink for printing each section was loaded into a 30 mL syringe (EFD Nordson). The printing temperature was set at 40°C. Herein, a stackable container with four sections was designed and printed using a fused deposition modeling (FDM) 3D printer (SOVOL 3D, Shenzhen Lian Dian Chuang Tech. LTD.) to facilitate the addition of support bath material during printing. Each section of the container had a length of 120.0 mm, width of 120.0 mm, and thickness of 15.0 mm, which could be stacked on top of each other. The photocurable yield-stress bath material was perfused into each section of the container at 4°C and left until the temperature was raised to 25°C. Then, the dispensing nozzle was moved within the thermosensitive yield-stress support bath to print the corresponding section of the contour layer. After the entire brain contour layer was printed, the container was moved to the refrigerator at 4°C for 1 h to thermally crosslink the contour layer and liquefy/recycle the support bath material outside the contour layer. After that, the contour-layered brain model was transferred to the CaCl_2_ bath at 4°C for 1 h for ionically crosslinking the contour layer. In the subsequent step, the UV curing system was applied for 20 min to photo crosslink the brain core within the contour layer. Finally, the contour layer was de-crosslinked and removed by submerging it in the sodium citrate bath at 37°C for 20 min. Thus, the brain core can be achieved, which was further and fully crosslinked by exposing it to UV light for 40 min.

For demonstration, a skull platform was fabricated using the FDM 3D printer to hold the full-scale human brain model. Then, the tumor lesion was removed from the brain model using a scalpel. Finally, the 3D brain patch was attached to the lesion to mimic the wound-healing process.

The full-scale brain model, brain patch model, and skull platform model were directly downloaded from GrabCAD (https://grabcad.com/) as STL files. For fabricating the brain, the brain model was further processed in Anycubic software (https://www.anycubic.com/) to be extracted as a shell structure with a thickness of 1.0 mm. The remaining models were designed using SolidWorks (Dassault Systemes SolidWorks Corp.) and saved as STL files. These files were sliced by the EnvisionTEC Perfactory software and imported into the 3D bioprinter for printing.

### 2.6 | Statistical analysis

All quantitative data in the text and figures were reported as means ± standard deviation (SD) with *n* = 3 samples per group.

## 3 | RESULTS AND DISCUSSION

### 3.1 | A set of yield-stress support bath-assisted 3D printing approaches

**FIGURE 1:**
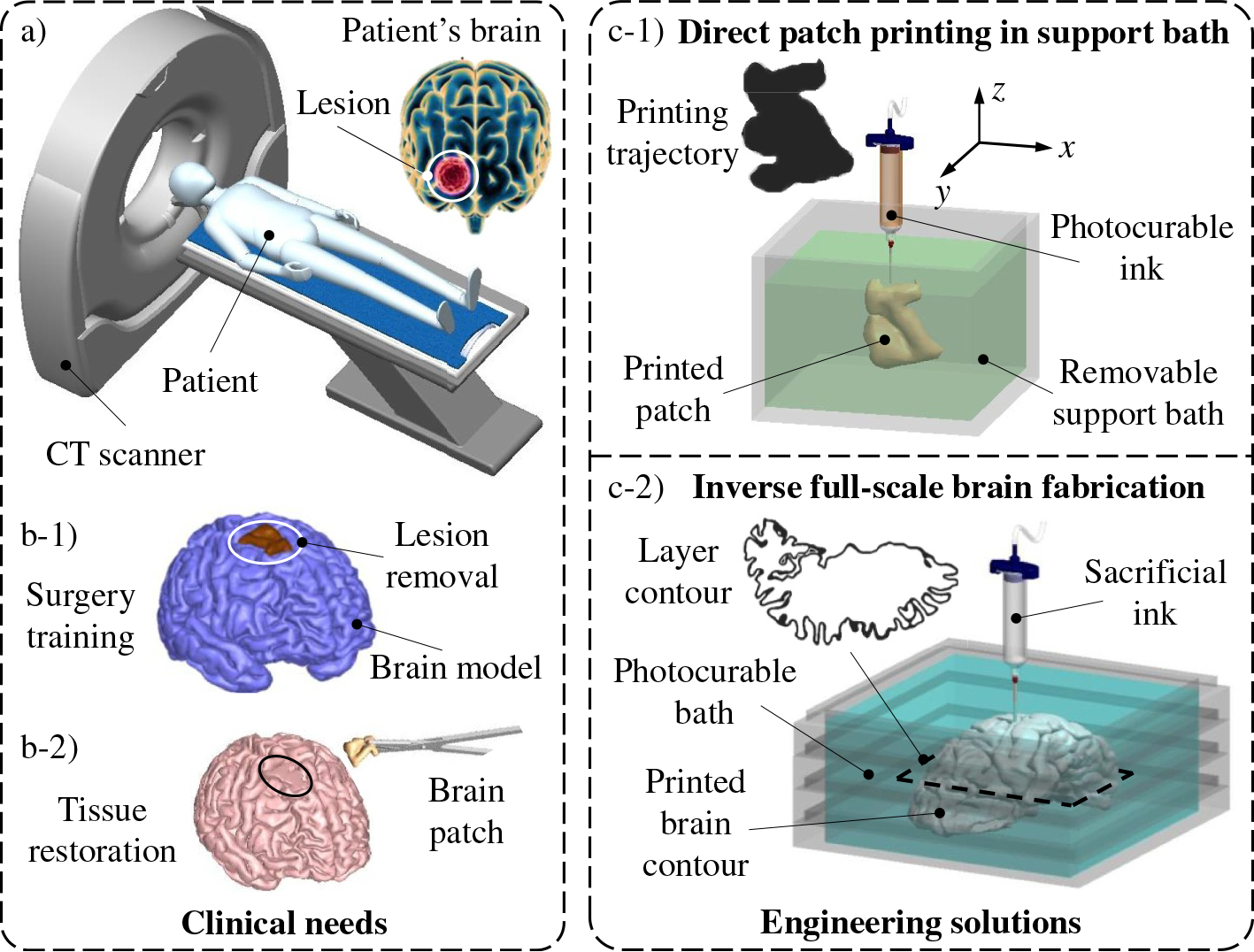
(a) CT scanning to obtain the geometrical data of the patient’s brain and lesion. Construction of (b-1) a full-scale human brain model for tumor surgery training and (b-2) a 3D brain patch to assist postoperative tissue restoration. Proposed 3D printing approaches: (c-1) stimuli-responsive yield-stress fluid-assisted 3D printing or “direct patch writing in support bath” and (c-2) inverse photocurable stimuli-responsive support bath-assisted 3D printing or “peeling-boiled-eggs” to fabricate the 3D brain patch and full-scale human brain model, respectively. 3D, three-dimensional; CT, computed tomography.

**FIGURE 2:**
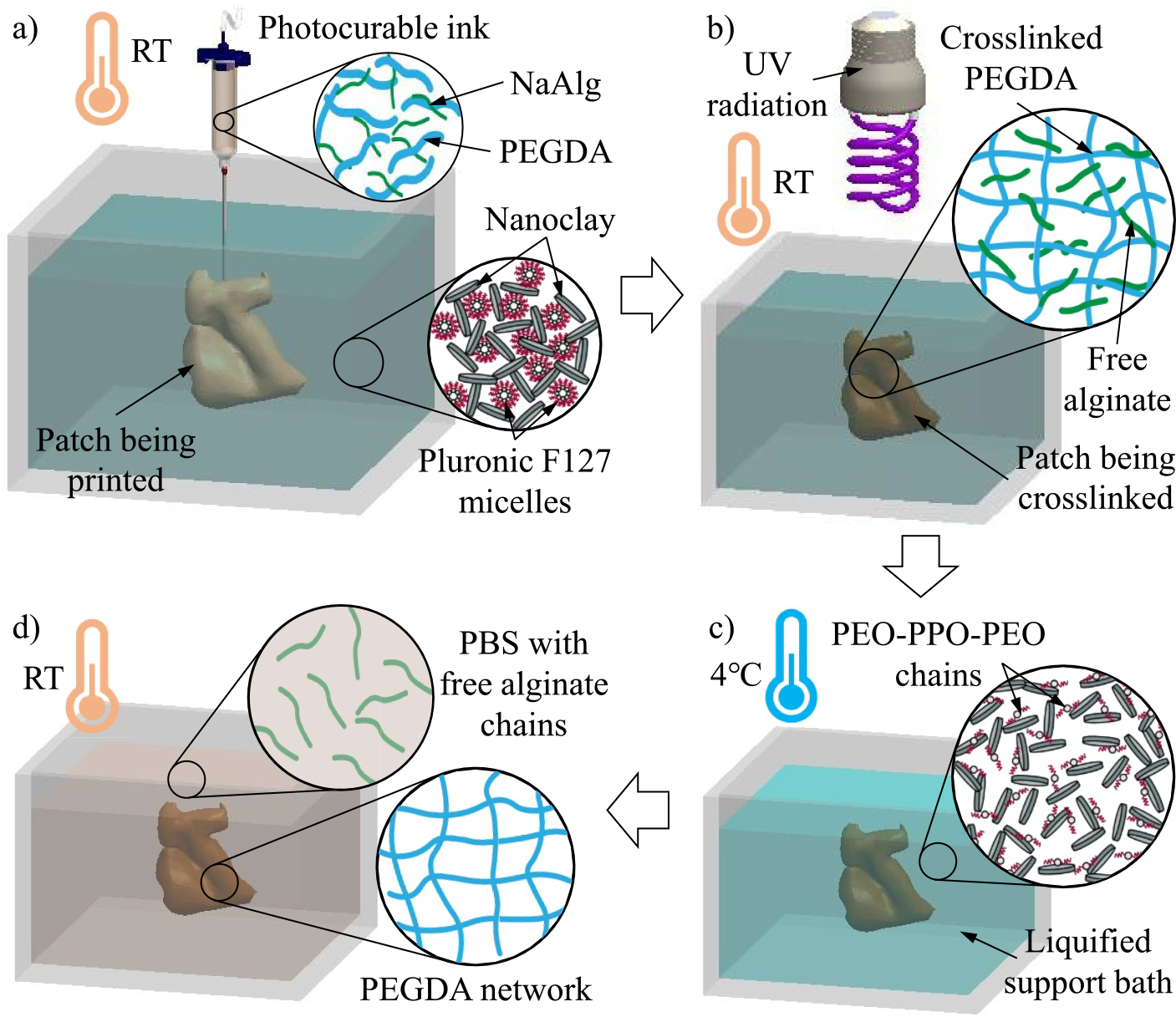
Mechanism of direct path writing in the thermosensitive yield-stress bath. (a) Photocurable ink printing in the yield-stress support bath at RT. (b) Photo crosslinking the printed path structure under UV radiation. (c) Liquefying the support bath at 4°C to release the crosslinked brain patch from the bath. (d) Removing alginate in the patch in the PBS bath at RT. PBS, phosphate-buffered saline; RT, room temperature; UV, ultraviolet.

This paper aims to develop a set of 3D printing approaches to fabricate customized full-scale engineered human brains for surgery training and specialized 3D brain patches for wound healing after surgery. The process involves performing CT scanning to obtain the geometric data of a patient’s brain and lesion as illustrated in Figure 1a, creating a digital 3D brain model, and duplicating an engineered patient’s brain with the lesion as shown in Figure 1b–1. Simultaneously, the lesion’s data is used to fabricate the customized brain patch, which perfectly fits the shape and size of the removed lesion (Figure 1b–2). Herein, two stimuli-responsive yield-stress fluid-assisted 3D printing approaches are developed and proposed. The first one is for brain patch printing, in which a photocurable ink is designed and used to print a 3D patch in a removable stimuli-responsive yield-stress support bath in a layer-by-layer manner (Figure 1c–1). After printing, UV radiation is applied to cause the chemical crosslinking of the patch. Then, an external stimulus, such as temperature change or pH variation, is performed on the support bath to tune its rheological behaviors. Thus, the patch can be easily released from the support bath, while the residual bath material on the patch surface is able to be rinsed mildly and completely. This approach is named “direct patch writing in support bath.”

For engineered full-scale human brain printing, an inverse fabrication approach, figuratively named “peeling-boiled-eggs (PBE),” is developed. In this approach, a sacrificial ink is adopted to only print the contour of each layer in a stimuli-responsive yield-stress support bath. Because the rheological properties of the bath material are tunable by the external stimuli, it is feasible to on-demand perfuse additional support bath(s) during printing to realize the creation of the contour layer of a full-scale human brain as reported in the previously published paper.^[30]^ Meanwhile, a photocurable hydrogel is added to the yield-stress fluid during preparation to make the support bath chemically crosslinkable under UV radiation. After printing, the contour layer from the sacrificial ink is solidified first using different crosslinking mechanisms to function as an egg shell and fully envelop the liquid brain core. The remaining support bath material outside the contour layer can be removed and harvested for the next fabrication cycle. After that, UV radiation is performed to photo crosslink the brain core through the solidified contour layer, like boiling an egg. Finally, the contour layer is de-crosslinked and liquefied using corresponding mechanisms (i.e., peeling a boiled egg) to release the brain core as the engineered full-scale human brain. Since only the contour layer is printed and then the enveloped core is crosslinked, this approach can realize low-waste and highly efficient manufacturing of human tissue/organ models in the future.

### 3.2 | Mechanism of direct patch writing in support bath

The working mechanism of the direct patch writing approach is illustrated in Figure 2. Herein, a thermosensitive yield-stress fluid is used as the support bath,^[30]^ which is composed of two components: Pluronic F127 and nanoclay additive. Pluronic F127 consists of two polymer phases—poly(ethylene oxide) (PEO) and poly(propylene oxide) (PPO)—that connect in an alternating linear PEO-PPO-PEO configuration. There is a critical micelle temperature $\left(T_{m}\right)$ that depends on the concentration of Pluronic F127. When the ambient temperature is above $T_{m}$, PEO-PPO-PEO species can form spherical micelles by surrounding a hydrophobic PPO core with a hydrophilic PEO corona.^[27,32]^ The aggregation of numerous micelles enables Pluronic F127 to present a solid-like state at the macroscopic level.^[33,34]^ When the ambient temperature is below $T_{m}$, the hydrophobic PPO core is hydrated to make individual PEO-PPO-PEO species soluble in an aqueous solvent,^[35,36]^ resulting in a solid-like-to-liquid transition of Pluronic F127. Nanoclay is an emerging 2D nanoadditive with positively charged edges and negatively charged faces.^[37,38]^ In an aqueous solvent, nanoclay platelets form a “house-of-cards” arrangement due to electrostatic interactions, which make the suspension to possess a yield-stress property for reversibly switching between liquid and solid-like states at different stressed conditions.^[22,29]^ The interactions between Pluronic F127 and nanoclay (inset of Figure 2a) enable the resultant nanocomposite not only to inherit the yield-stress property of nanoclay but also to possess the thermosensitivity of Pluronic F127. Therefore, it can serve as a thermosensitive support bath.^[30]^ The photocurable ink used for patch printing is also composed of two components: PEGDA and sodium alginate (NaAlg). PEGDA is chemically crosslinkable under UV exposure with the help of an initiator, but its precursor has a low viscosity. Thus, alginate is added to tune the ink’s viscosity and enhance printability.

Using this ink, a 3D brain patch can be printed in the support bath at RT, which is above $T_{m}$ (as shown in Figure 2a). After printing, UV radiation is applied to cause the photo crosslinking of the PEGDA component in the patch (Figure 2b). The ambient temperature is then lowered to below $T_{m}$ (e.g., 4°C). At such a low temperature, PEO-PPO-PEO species of Pluronic F127 lose their micelle morphology and become soluble in the aqueous solvent, leading to the liquefaction of the support bath (Figure 2c). As a result, the solidified brain patch can be easily released from the support bath and submerged in a PBS bath. Since the alginate component is not crosslinked in the patch, alginate molecules gradually diffuse from the patch to the PBS bath, resulting in the formation of the PEGDA brain patch with negligible alginate.

### 3.3 | Material characterization in direct patch writing

**FIGURE 3:**
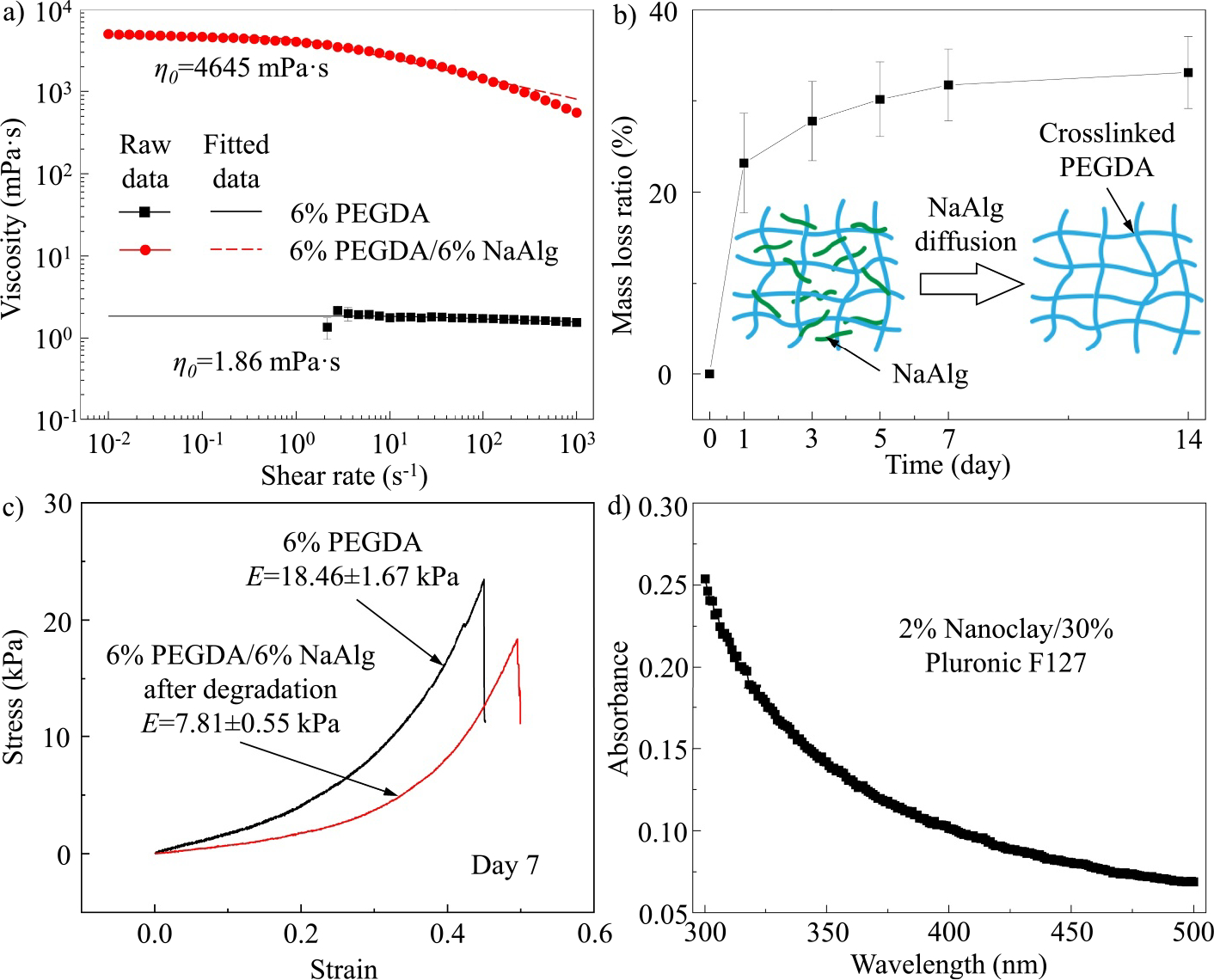
Material characterization in direct patch writing. (a) Viscosity as a function of the shear rate for photocurable inks with and without alginate. (b) Mass loss ratio of solidified PEGDA/NaAlg specimens as a function of degradation time. (c) Mechanical properties of pure PEGDA and PEGDA/NaAlg specimens after 7-day degradation. (d) UV transparency of nanoclay-Pluronic F127 yield-stress material. NaAlg, sodium alginate; PEGDA, polyethylene glycol diacrylate.

Since the thermosensitive yield-stress support bath has been well-demonstrated in the previous study,^[23]^ this work mainly focuses on the material characterization of photocurable ink, which must meet three basic requirements: (1) suitable viscosity for printing purposes, (2) acceptable degradation rate to serve as a stable patch after implantation, and (3) mechanical properties comparable with human brain tissues. Thus, the viscosities of the photocurable ink and pure PEGDA are measured first via the steady shear rate sweeps as shown in Figure 3a. Using the Carreau-Yasuda model: $\eta (\dot{\gamma })=\eta_{\infty }+\left(\eta_{0}-\eta_{\infty }\right){\left(1+(\lambda \dot{\gamma }{)}^{a}\right)}^{(n-1)/a}$ (where $\dot{\gamma }$ is the shear rate, λ is the relaxation time, n is the power law index, and a is the dimensionless parameter, and $\eta_{\infty }$ is the viscosity at an infinite shear rate that equates to zero in this case^[39]^), the zero-shear-rate viscosity $\left(\eta_{0}\right)$ can be determined. It is observed that although both materials are shear-thinning, $\eta_{0}$ of PEGDA with alginate is around 4645.00 ± 151.22 mPa·s, much higher than that of PEGDA without alginate (1.86 ± 0.41 mPa·s), which validates that the addition of alginate can effectively enhance the ink’s printability.

The degradation rate of photocurable ink after crosslinking is characterized by measuring the mass loss ratio of the PEGDA/alginate specimens in 14 days as illustrated in Figure 3b. It is found that the specimens lose their weights rapidly after submerging in a PBS bath for 1 day and then degrade gradually in the following 6 days. After 1 week, the variation of the mass loss ratio is almost negligible, which indicates that the patch made from the photocurable ink eventually maintains a stable morphology and mechanical properties for a relatively long time period. The dimensional variation of the specimens during this process is almost negligible. Since PEGDA presents a low degradation rate, as reported in literature,^[40–42]^ the pronounced increase of mass loss ratio on Day 1 is mainly attributed to the diffusion of alginate. Due to the high alginate concentration within the specimens, free-moving alginate chains can diffuse through crosslinked PEGDA networks into the PBS bath, leading to a decrease in the specimen weight, as shown in the inset of Figure 3b. Thus, after printing, the 3D brain patch needs to be submerged in the PBS bath for at least 7 days before use.

Diffusion of free alginate polymer chains also affects the mechanical properties. Herein, we assume that PEGDA has relatively stable properties during a 14-day degradation period, which has been reported in many pieces of literature.^[43–45]^ The stress-strain plots of pure PEGDA and PEGDA/alginate specimens after 7-day degradation are illustrated in Figure 3c. The diffusion of alginate results in the generation of extra voids within crosslinked PEGDA networks, which leads to the deterioration of mechanical properties. As seen from Figure 3c, the elastic modulus of PEGDA/alginate specimens is around 7.81 ± 0.55 kPa, which is lower than that of pure PEGDA (18.46 ± 1.67 kPa). However, this elastic modulus is close to the order of that of human brain tissues (dozens of kilopascals^[46,47]^), which validates that after degradation, the printed patch still meets the required mechanical properties.

Finally, the UV transparency of the nanoclay-Pluronic F127 nanocomposite is characterized as shown in Figure 3d. It is observed that in the wavelength range of UV light (300–500 nm), the absorbance of the nanocomposite is relatively low. Thus, UV light can effectively penetrate through the support bath without losing much energy to induce the photo crosslinking of the printed patch.

### 3.4 | Study on printing conditions

**FIGURE 4:**
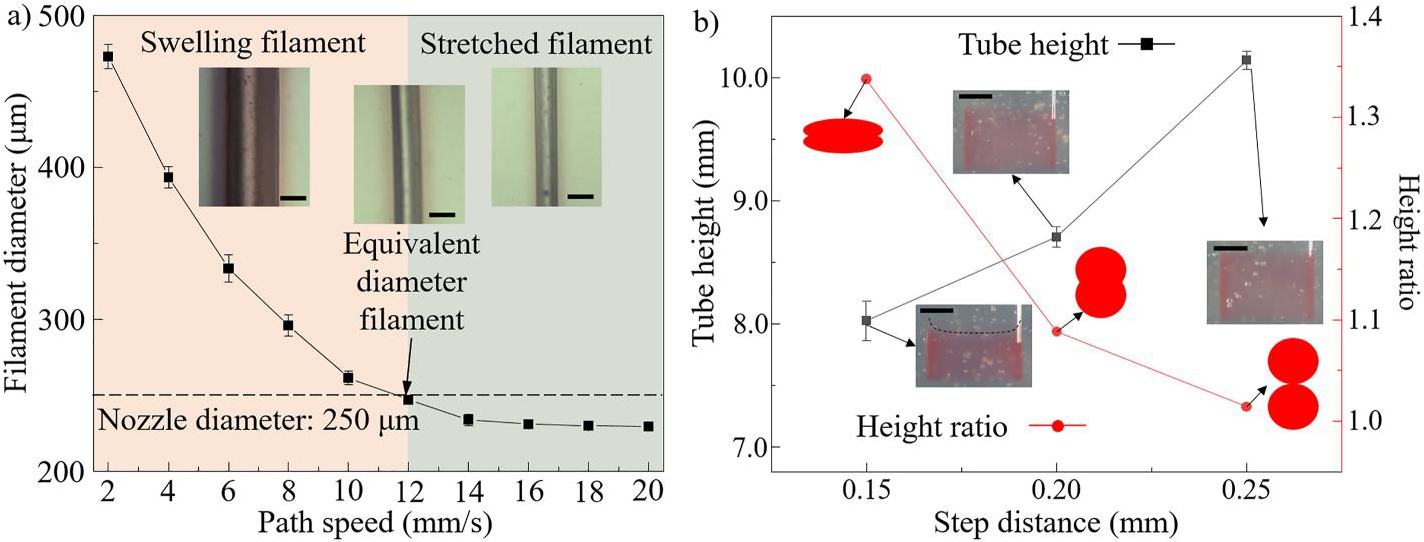
(a) Filament diameter and category as a function of path speed (scale bars: 250 μm). (b) Effects of step distance on tube height and height ratio (scale bars: 5 mm).

In support bath-assisted 3D printing, filament diameter and step distance are two main factors that affect the accuracy of a printed structure, and they are systematically investigated here. The filament diameter can be controlled by different printing parameters, such as dispensing pressure, nozzle geometries, and path speed.^[19,29]^ Among these parameters, path speed can be easily and dynamically adjusted to achieve filaments with a broader diameter range. As seen from Figure 4a, when the path speed increases from 2 to 20 mm/s, the filament diameter decreases from ~473 to 229 μm. Accordingly, the filament category changes from swelling filament with a diameter larger than the nozzle inner diameter to equivalent diameter filament with the size close to the nozzle diameter, and finally to stretch filament with a smaller filament diameter due to the dragging effects during printing, as shown in the insets of Figure 4a. The results are consistent with those in the published paper.^[20,29,30]^ Herein, a path speed of 12 mm/s is selected to generate filaments with a diameter of approximately 250 μm, which is equivalent to the inner diameter of the dispensing nozzle.

Step distance refers to the distance a printhead moves along the vertical direction to print adjacent layers. In this study, three step distances (i.e., 150, 200, and 250 μm) are set up to print straight cylindrical tubes to investigate their effects on tube height and height ratio as shown in Figure 4b and Movie S1. When the step distance (e.g., 150 μm) is much smaller than the filament diameter (~250 μm), an overdeposited phenomenon occurs, in which the dispensing nozzle is submerged by the previously extruded ink material. As a result, the printed tube height (8.03 ± 0.89 mm) exceeds the designed value (6.0 mm), leading to a height ratio of around 1.35. When the step distance (e.g., 250 μm) is close to the filament diameter, deposited filaments just come into contact with each other without any overlap as illustrated by the inserted schematic in Figure 4b. Although the printed tube height (10.16 ± 0.07 mm) in this scenario matches well with the designed height (10.0 mm), the entire tube lacks integrity when released from the bath because of the insufficient fusion between adjacent filament layers.^[22]^ When the step distance (e.g., 200 μm) is slightly lower than the filament diameter, inter-filament fusion can be enhanced during printing, while the overall tube height (8.71 ± 0.08 mm) is still controllable (designed height of 8.0 mm). Therefore, it is technically feasible to print well-defined structures with desired mechanical properties.

### 3.5 | 3D brain patch printing

**FIGURE 5:**
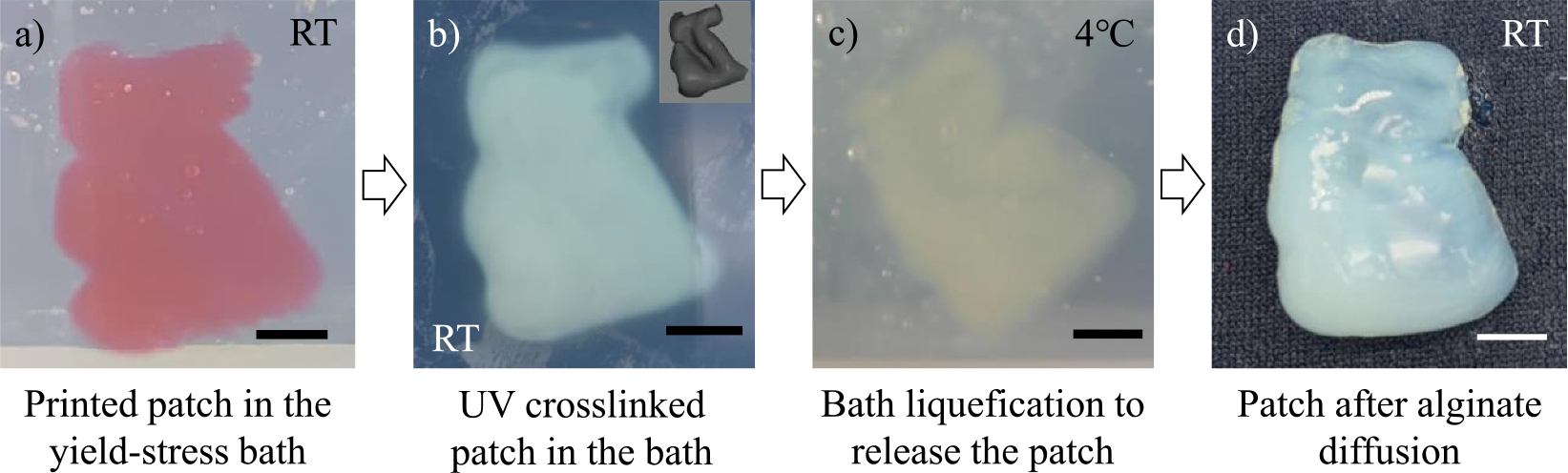
3D brain patch fabrication procedures: (a) liquid patch printing in the nanoclay-Pluronic F127 support bath at room temperature, (b) patch crosslinking in the bath via UV radiation, (c) releasing the crosslinked brain patch by liquefying the bath material at 4°C, and (d) 3D brain patch after alginate diffusion. Scale bars: 5 mm. 3D, three-dimensional; UV, ultraviolet.

After characterizing the ink properties and determining the suitable printing conditions, a 3D brain patch is printed in the nanoclay-Pluronic F127 yield-stress support bath at RT as shown in Figure 5a and Movie S2. The support bath can stably hold the liquid patch in situ before crosslinking. The support bath with the printed patch is then exposed to UV radiation. Due to excellent UV transparency, UV light can easily penetrate through the support bath material to cause the solidification of the PEGDA component within the patch as shown in Figure 5b. The crosslinked patch presents a well-defined shape that closely resembles the designed geometry (inset of Figure 5b). After crosslinking, the bath temperature is decreased to 4°C, at which Pluronic F127 rapidly switches from a paste state to a liquid state with good flowability.^[30]^ Thus, the patch could be readily harvested from the liquefied support bath (Figure 5c). The fabrication cycle, including 3D printing (~20 min) and post-processing (~40 min), is completed within approximately 1 h. Finally, the patch is submerged in the PBS bath for 7 days to let the alginate component fully diffuse out of the patch structure, leading to the creation of the 3D brain patch made from PEGDA, as shown in Figure 5d.

### 3.6 | Mechanism of the proposed “peeling-boiled-eggs” strategy

**FIGURE 6:**
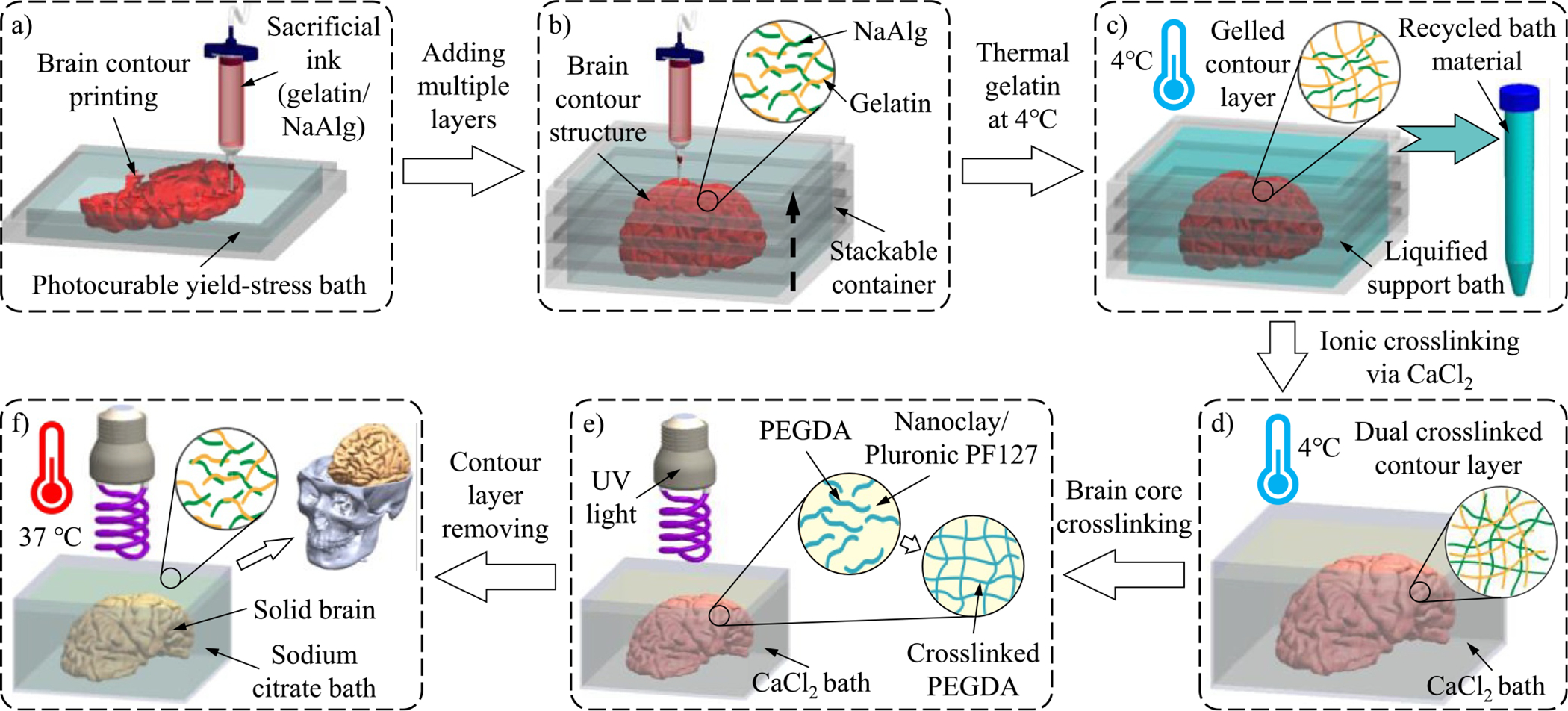
Mechanism of the PBE strategy. (a) Sacrificial ink (gelatin/NaAlg) printing in the photocurable yield-stress support bath to form the first section of the brain contour layer. (b) Brain contour structure after adding the photocurable yield-stress support bath in the stackable container several times. (c) Thermal gelation of the contour layer and liquefaction of the support bath at 4°C to recycle the bath material surrounding the brain contour layer. (d) Ionic crosslinking the contour layer in the CaCl_2_ bath to further enhance its mechanical stiffness. (e) Photo crosslinking the brain core with the contour layer under UV exposure. (f) Liquefying the counter layer in the sodium citrate bath at 37°C to release the solidified brain core. CaCl_2_, calcium chloride; NaAlg, sodium alginate; PBE, peeling-boiled-eggs; UV, ultraviolet.

The working mechanism of PBE is illustrated in Figure 6. PEGDA is added to the nanoclay-Pluronic F127 nanocomposite, which enables the on-demand perfusion of additional support bath material during contour layer printing. Additionally, PEGDA makes the brain core photo crosslinkable after the contour layer is solidified. The sacrificial ink is designed to have two main components: gelatin and NaAlg. Gelatin can be gelled by decreasing the ambient temperature and liquefied by increasing the temperature,^[21,48,49]^ while alginate is an ionically crosslinkable hydrogel that undergoes gelation when interacting with divalent ions (e.g., Ca^2+^) or trivalent ions (e.g., Al^3+^) to form a stable 3D network. Thus, the gelatin/alginate ink is printed in the photocurable yield-stress support bath to print the first section of the brain contour layer as shown in Figure 6a. After that, additional support bath material at 4°C is added into the next layer of the stackable container. At a low temperature, the bath material presents a low viscosity and good flowability to rapidly fill the container. When the bath temperature increases to RT, the nanocomposite changes from a low-viscosity liquid to a yield-stress fluid with rheological properties suitable to serve as a support bath. The printing process continues to print the next section of the brain contour layer. By repeating this contour printing-bath adding cycle, the entire brain contour layer can eventually be printed as illustrated in Figure 6b.

After printing, the gelatin component in the contour layer is gelled by decreasing the temperature to 4°C. At this temperature, the thermosensitive support bath loses its yield-stress property and switches to a liquid state with a low viscosity. Thus, the bath material outside the contour layer can be easily recycled for other printing applications, while the liquid brain core is completely enveloped by the contour layer without any leakage as illustrated in Figure 6c. To further enhance the mechanical stiffness of the contour layer, the printed structure is moved to a CaCl_2_ bath at 4°C to induce the ionic crosslinking of the alginate component in the contour layer as shown in Figure 6d. Then, the structure with a dual crosslinked contour layer is exposed to UV radiation. UV light can penetrate through the contour layer to cause the photo crosslinking of the PEGDA component within the brain core (Figure 6e). Finally, the solidified brain core, surrounded by the dual crosslinked contour layer, is submerged in a sodium citrate bath at 37°C. At this temperature, the gelatin component loses its networked microstructure and switches to the liquid state. Simultaneously, calcium ions in the calcium alginate network are replaced by the sodium ions provided by sodium citrate, causing the occurrence of de-crosslinking. As a result, the contour layer is fully liquefied to release the photo crosslinked brain, which is used as the full-scale human brain model, as shown in Figure 6f.

### 3.7 | Material characterization in the PBE strategy

**FIGURE 7:**
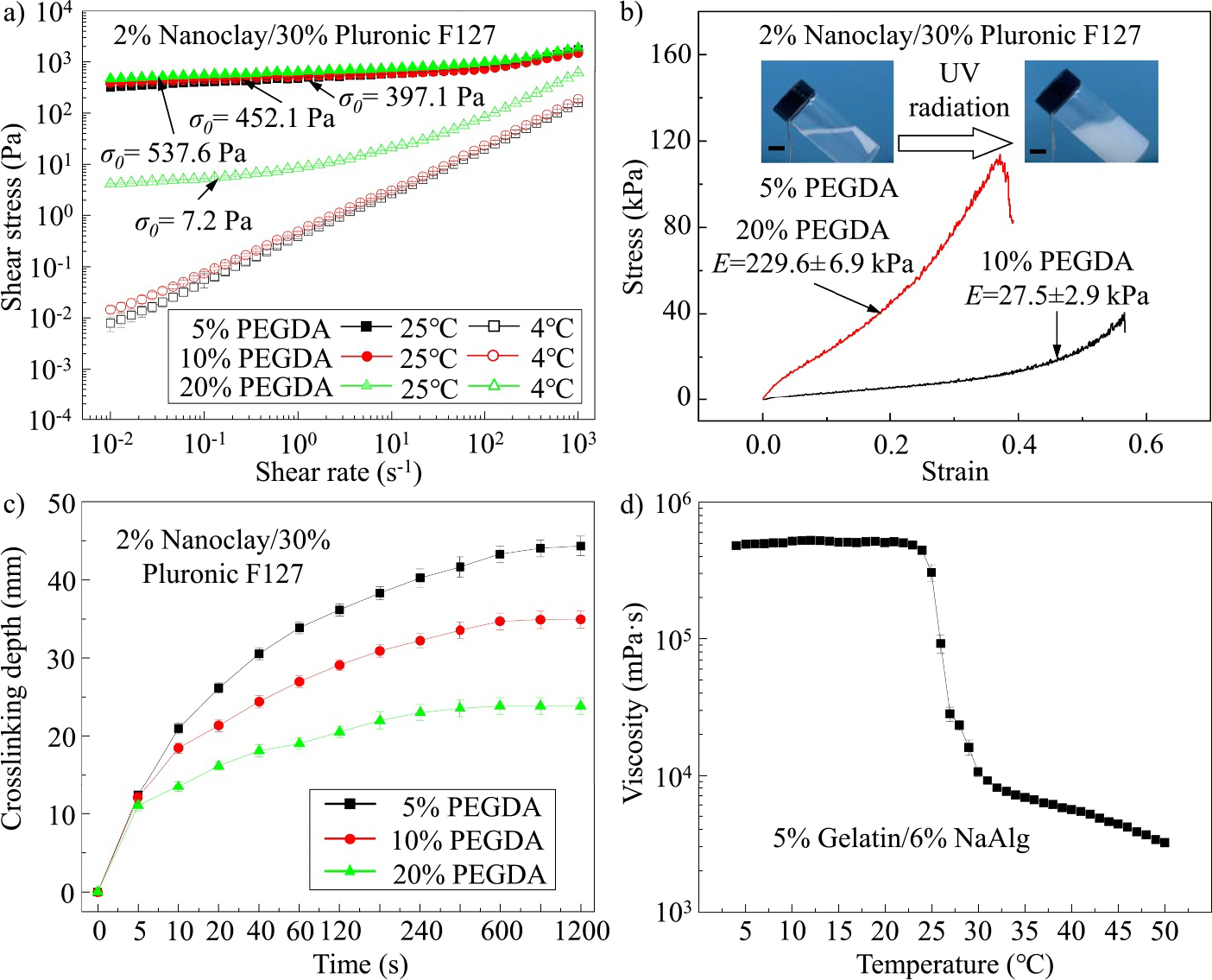
Material characterization in the PBE strategy. Effects of the PEGDA concentration on (a) rheological properties, (b) mechanical properties, and (c) crosslinking depth of the photocurable yield-stress support bath. (d) Viscosity of the sacrificial ink as a function of temperature. PBE, peeling-boiled-eggs; PEGDA, polyethylene glycol diacrylate.

Designing the photocurable support bath material and the sacrificial ink material is essential for realizing the proposed PBE strategy. First of all, the effects of PEGDA on the rheological properties of the support bath material are investigated. The basic formula of the bath material is the nanoclay-Pluronic F127 nanocomposite. The yield stress $\left(\sigma_{0}\right)$ at each PEGDA concentration is determined by fitting the data into the Herschel-Bulkley model,^[50]^ $\sigma (\dot{\gamma })=\sigma_{0}+k{\dot{\gamma }}^{n}$ where σ is the shear stress and k is the consistency index. As seen from Figure 7a, the addition of low concentrations of PEGDA (e.g., 5% and 10% (v/v)) leads to negligible effects on the rheological behaviors. At RT (i.e., 25°C), the photocurable support bath materials with 5% and 10% (v/v) PEGDA possess similar yield stress values (397.1 ± 7.6 vs. 452.1 ± 8.2 Pa) in the steady shear rate sweeps, while at 4°C, both materials lose their yield-stress property. However, when the PEGDA concentration is increased to 20% (v/v), the yield stress of the support bath material at RT increases slightly to 537.6 ± 6.5 Pa. However, the material also presents a relatively high yield stress (7.2 ± 0.3 Pa) at 4°C, which indicates that this photocurable support bath has poor flowability at low temperatures and cannot be added on-demand during printing and/or easily removed after printing. This phenomenon is mainly attributed to the interactions between nanoclay platelets and Pluronic F127 polymer chains at low temperatures. For the nanocomposite without PEGDA, when the temperature decreases to below $T_{m}$, Pluronic F127 morphology switches from jammed micelles to free-moving polymer chains with the alternating linear PEO-PPO-PEO configuration. PPO species attach to the surface of the nanoclay, making each nanoclay platelet surrounded by a PEO-PPO-PEO shell. The generation of the shell further isolates the electrostatic interactions among nanoclay platelets, which inhibits the formation of the “house-of-cards” arrangement. Therefore, the nanocomposite presents a low viscosity and good flowability at low temperatures. In contrast, PEGDA is a branched hydrogel.^[51–53]^ When its concentration exceeds a threshold value, numerous branched PEGDA polymer chains entangle free-moving PEO-PPO-PEO chains, disenabling the PPO attachment to nanoclay platelets and formation of PEO-PPO-PEO shells. As a result, the yield stress of the nanocomposite with 20% (v/v) PEGDA at 4°C is due to the generation of the “house-of-cards” arrangement of nanoclay platelets.

The addition of PEGDA also affects the mechanical properties of the photocurable support bath material after UV crosslinking as shown in Figure 7b. When the PEGDA concentration is relatively low, the support bath cannot be crosslinked. As illustrated by the insets of Figure 7b, the nanocomposite with 5% (v/v) PEGDA still shows a liquid state after long-term photo crosslinking. At higher concentrations, the support bath materials are crosslinkable, and the mechanical properties are sensitive to the concentration change. When the PEGDA concentration rises from 10% to 20% (v/v), the elastic modulus of the nanocomposite increases from 27.5 ± 2.9 to 229.6 ± 6.9 kPa. By comprehensively evaluating the rheological and mechanical properties, 10% (v/v) is finally selected as the PEGDA concentration to prepare the photocurable yield-stress support bath. After crosslinking, the elastic modulus is 27.5 ± 2.9 kPa, which is at the same order as that of human brain tissue (dozens of kilopascals), indicating that the engineered brain model meets the mechanical needs to mimic a real human brain. If living cells will be printed in the future, the effects of PEGDA on cell viability will be further characterized.

PEGDA concentration affects the crosslinking depth of printed structures under UV radiation. As shown in Figure 7c, when the PEGDA concentration increases from 5% to 20% (v/v), the maximum crosslinking depth decreases from 44.33 ± 1.25 to 23.84 ± 1.04 mm. This is because a denser crosslinked shell can be formed at a higher PEGDA concentration, which blocks the penetration of UV light through this layer to further crosslink the PEGDA component inside. Since the maximum crosslinking depth of the nanocomposite with 10% (v/v) PEGDA is around 34.93 ± 1.11 mm, close to half of the average maximum thickness of half a human brain (~35 mm^[54,55]^), a bidirectional crosslinking method can be used to ensure that the whole brain core can be fully crosslinked before removing the contour layer.

Finally, to determine the printing temperature and thermal gelation temperature, viscosity measurements of the sacrificial ink at different temperatures are measured as shown in Figure 7d. For the ink with 5% (w/v) gelatin and 6% (w/v) alginate, the viscosity of the ink is high and stable (~4.9 × 10^5^ mPa·s) in the temperature range of 5–25°C, indicating a gel state of the ink. When the temperature increases from 25 to 30°C, the viscosity decreases rapidly that demonstrates a gel-to-sol transition. Thus, in this work, the printing temperature is set at 40°C, at which the sacrificial ink has a relatively low viscosity of approximately 5.5 × 10^3^ mPa·s to facilitate the ink extrusion. After deposition, the ink can be quickly gelled in the support bath at RT to prevent ink material diffusion and fully crosslinked at 4°C after the entire brain contour is printed.

### 3.8 | Investigation of operating conditions during PBE

**FIGURE 8:**
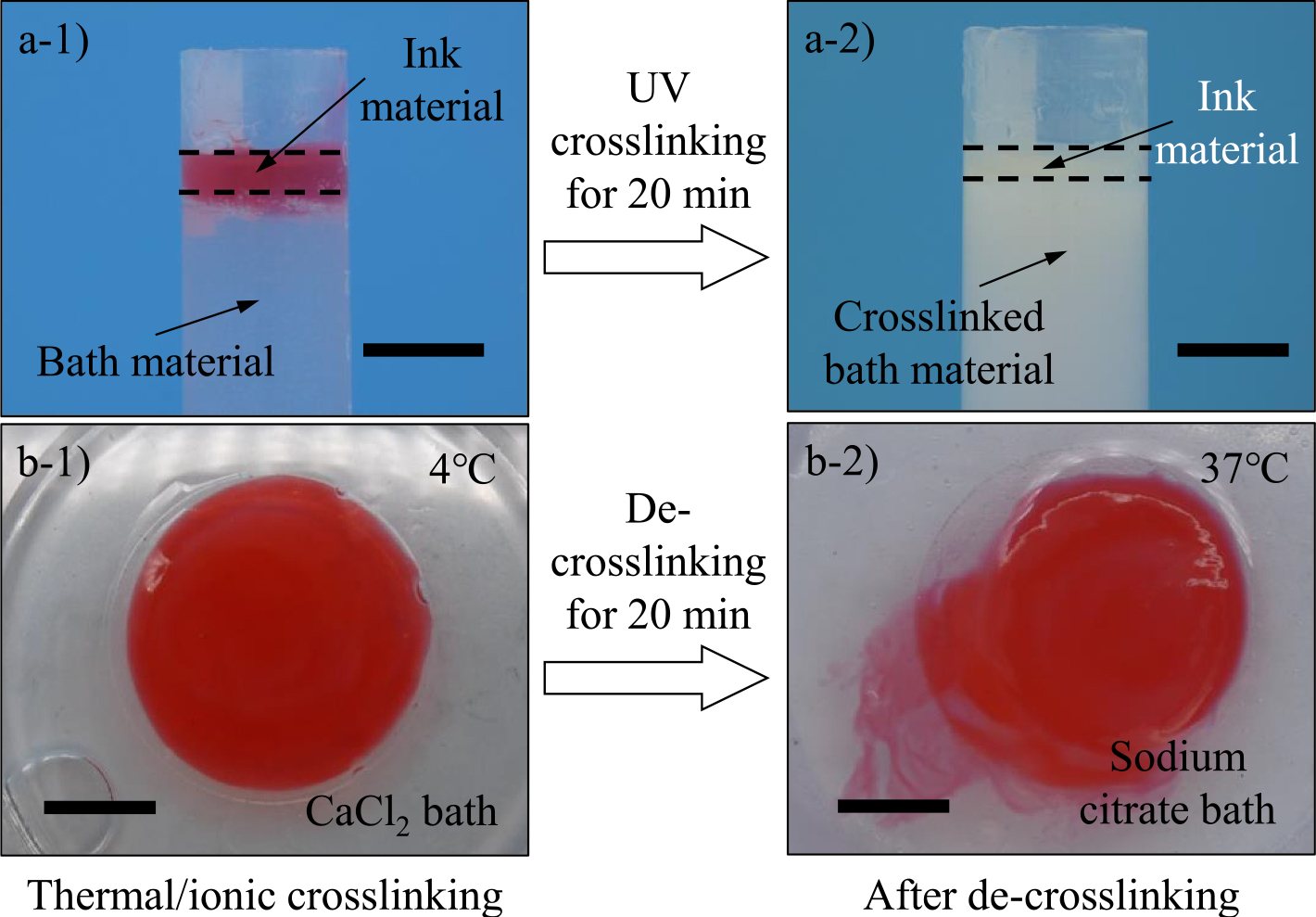
Support bath material underneath a solidified ink material layer (a-1) before and (a-2) after UV crosslinking. Thermally and ionically crosslinked specimens made from gelatin/alginate (b-1) before and (b-2) after de-crosslinking. Scale bars: 10.0 mm. CaCl_2_, calcium chloride; UV, ultraviolet.

Two key operating conditions must be determined before fabricating the full-scale human brain model, including (1) UV crosslinking time to fully solidify the brain core and (2) de-crosslinking time to completely remove the contour layer. As shown in Figure 8a, a crosslinking experiment is designed in which a gelled ink material layer with a thickness of 1.0 mm is atop the support bath material with a depth of 50.0 mm to mimic the scenario of the brain core covered by the contour layer (Figure 8a–1). UV radiation is applied vertically to penetrate through the ink material layer and photo crosslink the underneath support bath material. After 20 min, the support bath material is crosslinked with a depth of around 34.22 ± 0.16 mm as shown in Figure 8a–2. Thus, this time period is determined as the UV crosslinking time.

After that, the specimens made from the sacrificial ink are crosslinked in the CaCl_2_ bath at 4°C (Figure 8b–1) and then moved to the sodium citrate bath at 37°C. After 20 min, the solid specimens turn to a liquid state and start to spread in the bath as shown in Figure 8b–2. Therefore, the de-crosslinking time must be longer than 20 min to ensure the full liquefaction of the contour layer.

### 3.9 | Full-scale human brain fabrication via PBE

**FIGURE 9:**
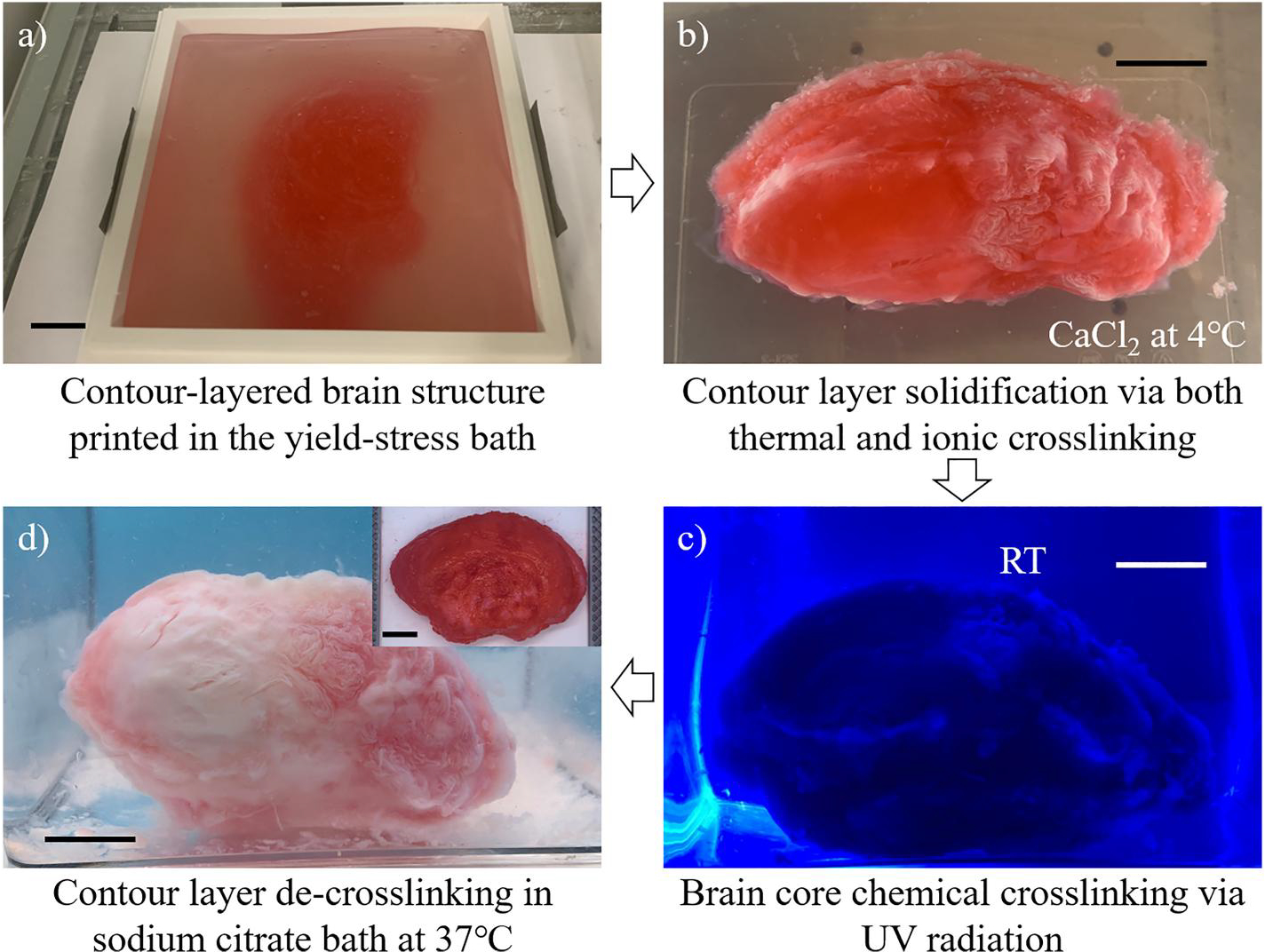
(a) First section of the contour layer printed in the photocurable yield-stress bath. (b) The contour-layered brain structure is thermally gelled and ionically crosslinked in the CaCl_2_ bath at 4°C. (c) Brain core photo crosslinking under UV radiation. (d) De-crosslinking of the contour layer in the sodium citrate bath at 37°C and released brain core (inset). Scale bars: 20 mm. CaCl2, calcium chloride; UV, ultraviolet.

A full-scale human brain model is fabricated after understanding the material properties and determining the key operating conditions. Due to the travel range of the 3D printer, an infant brain model is created. As shown in Figure 9a, the bottom section of the brain contour layer is printed in the photocurable yield-stress support bath. Then, additional support bath material at 4°C is added into the stackable container to fabricate the rest of the contour layer section by section as shown in Movie S3. The total printing period is around 5 h. The success rate of 3D printing is affected by many factors, such as material stability, printer status, printing parameters, environmental conditions, etc. By carefully selecting the materials and systematically studying the printing conditions, one-half of the human brain model is successfully printed. After printing, the support bath is moved to a refrigerator to thermally gel the contour layer and simultaneously liquefy the support bath material outside the contour layer. Then, the gelled contour layer enveloping a liquid brain core is submerged in the CaCl_2_ bath at 4°C to ionically crosslink alginate in the contour layer as shown in Figure 9b.

After that, the dual crosslinked contour structure is exposed to UV radiation, as shown in Figure 9c, for photo crosslinking the brain core. Twenty minutes later, the structure is flipped over to crosslink from the opposite direction. Thus, the entire brain core can be fully crosslinked. To mildly remove the contour layer, the structure is finally submerged in the sodium citrate bath at 37°C. The temperature increase and the existence of sodium ions result in the simultaneous de-crosslinking of gelatin and alginate networks. Therefore, more than 20 min later, the brain core without the contour layer is harvested from the bath. These post-printing procedures can be seen in Movie S4. If the brain core is not completely cured, additional UV radiation can be performed after removing the contour layer. The final half human brain model is shown in the inset of Figure 9d.

### 3.10 | Demonstration of full-scale human brain model with 3D printed patch

**FIGURE 10:**
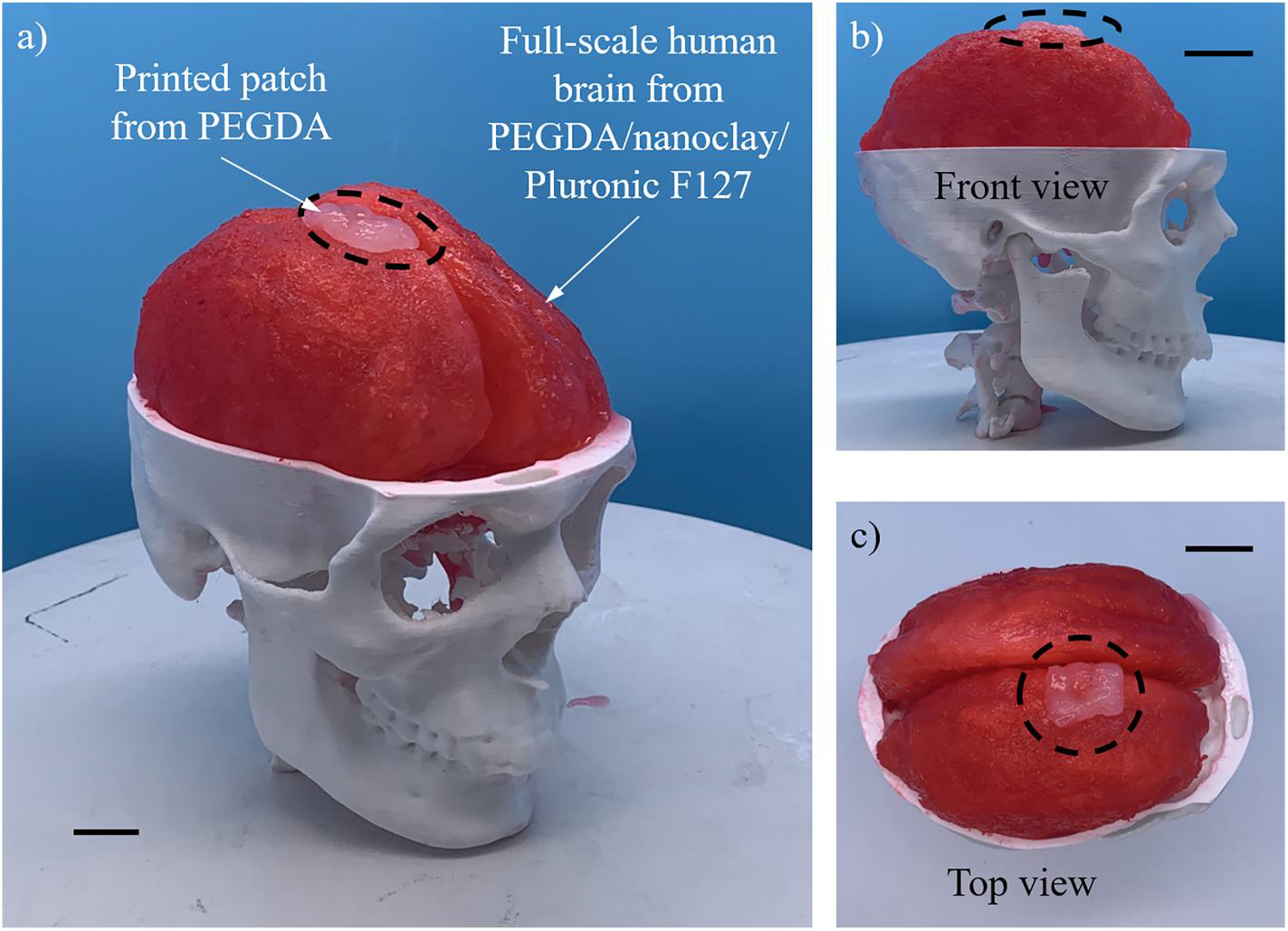
Engineered full-scale human brain model with the three-dimensional patch in a mock skull structure: (a) global, (b) front, and (c) top views. Scale bars: 20 mm.

The same printing protocol used for the first half of the human brain model is repeated to fabricate the other half. A mock tumor is then removed from the brain’s surface using a scalpel based on the lesion morphology and shape to mimic the surgery training process. Finally, the printed patch is attached to the lesion location to mimic the wound healing process. The brain model with the 3D patch is illustrated in Figure 10 and Movie S5, which validates the effectiveness of the proposed 3D printing approaches to fabricate engineered full-scale human tissues and organs for surgery training as well as customized 3D patches for different wound healing. It is noted that a handful of studies have been reported regarding the fabrication of full-scale human brain models. For example, Wagner et al.^[56]^ used inkjet printing to print soft brain tissues from a commercial-based material called “Tango+.” Dho et al.^[57]^ applied FDM to fabricate a polymer brain model. However, the build materials used in these studies are immiscible with living cells, so the proposed 3D printing approaches are not expandable for creating cellular human brains. To create brain prototypes from cytocompatible hydrogels, Ploch et al.^[58]^ developed a 3D printing-assisted two-step casting strategy. In this strategy, FDM was used to print a polymer brain model that was cast over with a silicone rubber. After the first-step casting, the model was dissolved, resulting in the formation of the silicone rubber mold for the second-step casting of the gelatin solution. When gelatin was thermally crosslinked, the silicone rubber mold was removed to release the gelatin brain model that can mimic the strength of human brain tissues. However, this strategy requires skillful and well-equipped operators to complete multiple complex procedures and is time-consuming with a total fabrication cycle of around 17 h, making it not applicable for clinical applications. Compared to these previously published methods, the set of 3D printing approaches in this work is advantageous and promising for the fast and accurate fabrication of full-scale human tissues and organs with complex anatomical architectures in the future. In addition, photocurable support bath materials for the PBE strategy can be partially recycled, reducing the fabrication cost and making it possible to massively produce human tissue/organ models for surgery training and other applications.

Although living cells are not involved in this work, the biocompatibility of the proposed 3D printing approaches is theoretically desirable. Cell viability in 3D printing is directly determined by two factors: (1) materials and (2) fabrication procedures. Herein, the materials include ink materials, support bath materials, and reagents in the post-printing processes. For ink materials, alginate,^[29,59]^ PEGDA^[60,61]^/photoinitiator,^[60,62]^ and gelatin^[21,63]^ are three representative biomaterials that have been widely used for diverse biomanufacturing and biomedical applications. Their excellent biocompatibility has been well investigated and validated. Nanoclay and Pluronic F127 are the two main components of the support bath materials. Nanoclay is a bioactive material that has been used as a carrier to control the release of drugs, growth factors, and proteins for cells.^[64–66]^ Pluronic F127 is one of the common components in cell culture mediums that can promote cell adhesion during culturing.^[67,68]^ The reagents used in the PBE approach are CaCl_2_ for ionically crosslinking alginate and sodium citrate for de-crosslinking alginate components. Both of them have negligible effects on cell growth and differentiation if their concentrations are carefully selected.^[69–75]^ In this work, the concentration of CaCl_2_ is 2.0% (w/v), which has been used for the 3D printing of living cells with good viability.^[76]^ Sodium citrate buffer has also been applied for bioprinting applications that can achieve over 90% cell survival rate.^[72–75]^ Therefore, considering the materials, the set of 3D printing approaches should have acceptable cell viability if living cells are printed. For the fabrication procedures, UV crosslinking period,^[77–80]^ crosslinking/liquefaction temperatures^[81]^ in PBE, and immerging periods in CaCl_2_ and sodium citrate baths^[71]^ all have controllable effects on cell viability^[82,83]^ if cell-laden bioinks are utilized, which will be further investigated in the future to alleviate cell damage during fabrication. However, if cells are seeded on the crosslinked patch or brain model, the negative effects of both materials and fabrication procedures can be neglected. It is reported that porous microstructures are formed within printed structures from PEGDA and Pluronic F127^[84,85]^ to allow cell inoculation. Thus, this cell seeding strategy is technically promising.

Moreover, the proposed PBE strategy offers the potential for constructing a heterogenous brain model with embedded cerebral vessels and cranial nerves. Because the photocurable support bath material is a yield-stress fluid, it is technically feasible to print 3D patterns from both a sacrificial ink and a cell-laden ink in the support bath via e-3DP before printing the contour layer. After crosslinking the brain core and removing the contour layer, the 3D pattern from the sacrificial ink can be rinsed through additional post-treatments to form hollow networks, serving as the cerebral vessels inside the brain, while the cellular 3D pattern can be encapsulated in the brain to function as the cranial nerves. Thus, a full-scale human brain model with anatomical features can be accurately duplicated.

In this study, the brain patch model is generated based on the brain CT data of a patient before surgery. From the clinical application standpoint, the deformation of brain tissue after surgical resection of a tumor can result in the misfit between brain tissue and the printed patch. Therefore, future brain patch models with arbitrary shapes should be created according to the CT data immediately after surgery to ensure that printed patches have a high shape accuracy and fitting capability. The high printing speeds will make the fast fabrication of customized brain patches technically feasible.

## 4 | CONCLUSIONS AND FUTURE WORK

In this work, a set of yield-stress support bath-assisted 3D printing approaches, including direct patch writing and PEB strategy, is systematically investigated. The material design and printing issues are discussed in detail. Finally, the full-scale human brain model as well as the 3D patch are successfully fabricated to mimic the surgery training and wound healing processes.

This work is a proof-of-concept study that validates the proposed set of 3D printing approaches. Future work will focus on the following aspects. (1) More in vivo research will be performed to investigate the mechanical strength change of printed models, possible immune rejection, efficacy of postoperative recovery after patch implantation, etc. In addition, drug-loaded 3D patches will be printed, which will be used to controllably deliver drugs to wounds with complex geometries to study the in vivo healing rate. (2) Improving the accuracy of fabricated models is another research direction. In 3D printing, many process parameters, such as nozzle diameter, layer height, path speed, crosslinking time, and so on, affect the final resolution and shape fidelity. To achieve high-precision, full-scale human brain models with sulci and gyri, these parameters will be comprehensively evaluated and optimized. In addition, the deformation of soft materials during post-treatments is always a challenge that can significantly affect the accuracy of final products. The corresponding operations need to be improved to avoid any undesired deformations. (3) From the clinical application perspective, neurosurgeons will be invited to assess the functionality of the brain models for surgery training by performing simulated surgical procedures as well as discuss the effects of model accuracy on the prognosis and functional recovery of brain tissues after surgery. (4) Manufacturing-wise, heterogeneous brain models with both vascular and neural networks will be printed by developing more innovative 3D printing-assisted methods based on this work. Furthermore, more detailed brain sections^[86]^ (such as telencephalon, diencephalon, brain stem, and cerebellum) as well as other full-scale human tissues and organs (such as the cornea, eyeball, and heart) will be manufactured using the proposed set of 3D printing approaches to eventually determine the printing capability space of these approaches. Finally, the study of how to automate the fabrication processes is of great significance from the clinical application aspect, which will be studied in the future.

## Supplementary Material

Movie S1_Step distance effects

Movie S2_Patch printing

Movie S4_Post printing treatments

Movie S5_Printed brain with patch

Movie S3_Brain printing

## Data Availability

All data used to support the findings of this study is included within the article. Raw data used to generate the figures are available from the corresponding author upon request.

## Key points

### What is already known about this topic?

- Current three-dimensional (3D) printing and derived approaches can only create scaled-down human brain architectures from biocompatible materials or full-scale brain structures from resins.

### What does this study add?

- The proposed embedded ink writing strategy can directly print specialized 3D brain patches in a stimuli-responsive yield-stress bath for wound healing after surgery.
- The newly developed inverse fabrication strategy, figuratively named “peeling-boiled-eggs,” realizes the efficient construction of biocompatible full-scale human brain models via 3D printing that can be used for surgery training and other brain-relevant studies.
